# An Open‐Label Randomized Controlled Trial Comparing the Efficacy and Safety of a 7‐Day Triple Therapy With Bismuth Versus 14‐Day Standard Triple Therapy for *Helicobacter pylori* Eradication in Children and Adolescents

**DOI:** 10.1111/hel.70103

**Published:** 2026-01-07

**Authors:** Anja Šterbenc, Bor Vratanar, Eva Miler Mojškerc, Matjaž Homan

**Affiliations:** ^1^ Institute of Microbiology and Immunology, Faculty of Medicine University of Ljubljana Ljubljana Slovenia; ^2^ Institute for Biostatistics and Medical Informatics, Faculty of Medicine University of Ljubljana Ljubljana Slovenia; ^3^ Department of Pediatrics Slovenj Gradec General Hospital Slovenj Gradec Slovenia; ^4^ Department of Gastroenterology, Hepatology, and Nutrition, University Children's Hospital, Faculty of Medicine University of Ljubljana Ljubljana Slovenia

**Keywords:** bismuth, children, eradication, *Helicobacter pylori*, treatment

## Abstract

**Background:**

To achieve eradication rates > 90%, the ESPGHAN/NASPGHAN guidelines for pediatric *Helicobacter pylori* infection recommend tailored antimicrobial therapy using sufficiently high doses over 10–14 days. However, prolonged treatment often leads to suboptimal compliance in children, which is a major contributor to reduced eradication rates. To address this, we evaluated the efficacy and safety of a shorter, 7 day triple therapy with bismuth compared with the 14 day standard triple therapy without bismuth in *H. pylori* infected children.

**Materials and Methods:**

From 2020 to 2024, we carried out a randomized controlled trial involving treatment‐naïve children and adolescents (5–18 years old) with confirmed *H. pylori* infection. Eligible participants were randomly allocated to receive either a 7 day triple therapy with bismuth (bismuth subcitrate, a proton pump inhibitor [PPI], amoxicillin, plus clarithromycin/metronidazole) or a 14 day standard triple therapy (a PPI, amoxicillin, plus clarithromycin/metronidazole) without bismuth. Two months after completing therapy, treatment success was determined using either a two‐step monoclonal stool antigen assay or a urea breath test. Any adverse events were documented using a structured questionnaire.

**Results:**

Seventy‐three children were enrolled in the study. In the intention‐to‐treat analysis, eradication was achieved in 91% of children treated with the 7 day triple therapy with bismuth and 87% of those receiving the 14 day standard triple therapy (*p* = 0.695). Per‐protocol eradication rates were 94% and 87%, respectively (*p* = 0.418). No serious adverse events were reported, and most adverse events were mild to moderate. A metallic taste was significantly more frequent in the 14 day standard triple therapy group, while other adverse events occurred with similar frequency.

**Conclusions:**

Adding bismuth to a 7 day triple regimen achieved high eradication rates and a safety profile similar to 14 day standard triple therapy, supporting its use as an effective and safe treatment option for pediatric *H. pylori* infection.

## 1 Introduction

Despite a marked decline in global incidence and prevalence in recent years, *Helicobacter pylori* remains among the most common bacterial infections in humans [1]. Notably, while the global crude prevalence in adults declined from 52.6% before 1990 to 43.9% between 2015 and 2022, no such trends were observed in pediatric or adolescent populations, with the prevalence in children/adolescents during 2015 to 2022 still as high as 35.1% [2].

In the 1990s, triple therapy, consisting of a proton pump inhibitor (PPI) and antibiotics, including amoxicillin and either clarithromycin or nitroimidazole, was introduced for the eradication of *H. pylori* infection. However, over the past decade, eradication rates with this regimen have fallen (primarily because of antimicrobial resistance), prompting recommendations for increased drug dosages, extended treatment duration, or the use of regimens containing three rather than two antibiotics, administered sequentially or concurrently [3].

Adverse events such as abdominal pain, nausea, taste disturbances, and diarrhea are commonly reported during *H. pylori* eradication therapy in children [4, 5]. Especially extended treatment durations recommended also in recent guidelines may augment this problem, potentially leading to early discontinuation of therapy [6]. This is particularly concerning because non‐adherence to therapy is recognized as the most common cause of *H. pylori* treatment failure in patients where treatment was prescribed according to the antibiotic susceptibility testing. Thus, strategies aimed at improving compliance in children—particularly by reducing adverse events—may enhance eradication rates and help limit the emergence of antibiotic‐resistant strains [7].

Although bismuth salts have been used in *H. pylori* treatment for more than three decades, their use diminished with the rise of PPI‐based triple therapy. Recently, however, an increase in resistant *H. pylori* strains and declining eradication rates have led to renewed interest in bismuth‐based regimens, due to bismuth's synergistic effects when combined with antibiotics, which may help overcome resistance to certain antimicrobials, including clarithromycin or metronidazole [4, 8]. Thus, bismuth subsalicylate or subcitrate has been included as a component of first‐line *H. pylori* eradication therapy in children in both the 2011 [9], and 2017 [10], pediatric guidelines issued by the European and North American Societies for Pediatric Gastroenterology, Hepatology and Nutrition (ESPGHAN/NASPGHAN). The most recent ESPGHAN/NASPGHAN guidelines advise that, in the absence of antimicrobial susceptibility data, a 10–14‐day bismuth‐based regimen should be used as the empiric first‐line treatment for *H. pylori* infection in pediatric and adolescent patients [6]. However, bismuth salts are rarely used in clinical practice, largely due to their unavailability in pharmacies across many European countries. While there is substantial evidence supporting their use in adults, prospective data on treatment protocols and eradication rates involving bismuth salts in the pediatric population remain scarce.

Thus, the objective of this study was to assess and compare the safety and efficacy of two eradication regimens for *H. pylori* in pediatric patients: a shorter, 7 day triple therapy with bismuth and a 14 day standard triple regimen without bismuth.

## 2 Materials and Methods

### 2.1 Study Population

This study was conducted between October 2020 and August 2024. Consecutive pediatric patients presenting with dyspeptic symptoms and referred for esophagogastroduodenoscopy (EGD) at one of three participating centers from Slovenia were considered for enrollment. Eligibility criteria included age between 5 and 18 years, body weight exceeding 15 kg, absence of prior treatment for *H. pylori* , and microbiological confirmation of *H. pylori* infection, including successful culture and susceptibility testing.

Exclusion criteria included: severe acute or chronic gastrointestinal disorders such as inflammatory bowel disease or coeliac disease, or other organic conditions that could interfere with symptom evaluation; presence of gastrointestinal ulcers; use of immunosuppressive agents, corticosteroids, or nonsteroidal anti‐inflammatory drugs (NSAIDs); use of PPIs within the 2 weeks prior to EGD; treatment with bismuth salts or antibiotics within the preceding 4 weeks; infection with an *H. pylori* strain resistant to both clarithromycin and metronidazole; known hypersensitivity to any study medication; and inability to swallow tablets or capsules.

### 2.2 The BismoHelP (BHP) Protocol

The trial was designed as a prospective, open‐label randomized controlled study. At the initial visit, an EGD was performed, and two gastric biopsies—one from the antrum and one from the corpus—were obtained for *H. pylori* culture. Additional biopsies were taken for rapid urease testing and histopathology evaluation according to the updated Sydney System protocol [11]. At the follow‐up visit scheduled 14 days after the EGD, when the results of antibiotic susceptibility testing were available, eligible patients were invited to participate in the study. In addition, written materials in Slovenian, freely available on ESPGHAN websites and addressing the importance of compliance with eradication therapy, were provided to the parents (https://www.espghan.org/knowledge‐center/education/H‐Pylori‐Patient‐Parent‐Guide). Written informed consent was obtained from all participants and/or their parents.

Participants were randomly assigned to the treatment groups by drawing sealed envelopes containing a card labeled either “K” or “T” (control vs. therapeutic group). The therapeutic group received a 7 day therapy consisting of a PPI, amoxicillin, either clarithromycin or metronidazole, and bismuth subcitrate, while the control group received a 14 day standard triple therapy comprising a PPI, amoxicillin, and either clarithromycin or metronidazole, prescribed according to the antibiotic susceptibility testing.

Drug dosages were adjusted according to body weight, based on the recommendations by Jones et al. [10] For the group receiving triple therapy with bismuth, the regimen included colloidal bismuth subcitrate 120–240 mg twice daily, a PPI 20–40 mg twice daily and antibiotic therapy, tailored to antibiotic susceptibility testing: amoxicillin 500–1000 mg two times daily, and either metronidazole 250–500 mg twice daily or clarithromycin 250–500 mg twice daily. This combination was administered for 7 consecutive days. Except for bismuth subcitrate, the standard triple therapy regimen was identical and administered over a 14 day period.

To monitor treatment adherence and document adverse events, each participant was provided with a diary upon enrollment. The diary (covering either 7 or 14 days, depending on the assigned treatment) captured daily information on symptoms including abdominal pain, bloating, nausea, metallic taste, vomiting, bowel movements, and any notable events. Symptom severity was recorded in patient diaries using a 0–3 scale (0 = none, 1 = mild without interference in normal activity, 2 = moderate with interference in normal activity, 3 = severe, preventing daily activity). Stool frequency and consistency were assessed according to the Bristol Stool Scale (BSS) as follows: 0 = no stool, 1 = hard (BSS 1–2), 2 = formed (BSS 3–4), 3 = soft (BSS 5–6), and 4 = watery (BSS 7). Diarrhea was defined as more than two soft stools (BSS 5–6) in a day and/or at least one watery stool (BSS 7), while constipation was defined as fewer than three bowel movements per week and/or stools of hard consistency. A study nurse contacted participants by phone to ensure consistent data entry. In addition, any deviations to the drug intake and use of concomitant medications were recorded. Treatment adherence was evaluated during the follow‐up visit. Full adherence to the protocol was considered as per‐protocol (PP), whereas any deviations to the protocol were considered as protocol violations and these patients were included in the intention‐to‐treat (ITT) analysis only.

*H. pylori* strain culture and antimicrobial susceptibility testing was performed from gastric biopsies, as described in detail previously [12].

Assessment of *H. pylori* eradication was conducted 2 months after completion of therapy, using either the 13C‐urea breath test or LIAISON Meridian *H. pylori* SA assay (DiaSorin Inc., Stillwater, MN, USA), a highly reliable two‐step stool antigen test employing monoclonal antibodies.

Ethics Statement for the study was obtained from the Ministry of Health of the Republic of Slovenia (approval number 0120–91/2020/6), and the trial was registered on ClinicalTrials.gov (identifier: NCT06143124). Participant anonymity was maintained through coding.

## 3 Statistical Analysis

Before recruitment, we performed an a priori power analysis to compare *H. pylori* eradication rates between the two treatment groups. We assumed an eradication rate of 85% for the 14 day standard triple therapy and 95% for the 7 day triple therapy with bismuth. Based on these assumptions, a total sample size of 281 participants (two‐sided α = 0.05, 80% power) would be required to detect a statistically significant difference. However, due to the low prevalence of *H. pylori* infection in our pediatric population (10.9%) [13], and the limited number of eligible patients in Slovenia, achieving this sample size within a reasonable timeframe was not feasible. The study was therefore conducted pragmatically, including all eligible patients who presented during the recruitment period.

For the *H. pylori* eradication outcomes, analyses were conducted on both the ITT and PP populations. Fisher's exact test was used to compare eradication rates between the 7 day triple therapy with bismuth and the 14 day standard triple therapy. The estimated eradication proportions with 95% confidence intervals (CIs) calculated by the Wilson method and corresponding risk difference and odds ratios (ORs) were reported. A similar approach was applied to the PP analysis, which included only participants who fully adhered to the protocol.

For the adverse events analysis, only participants with full adherence were included. To facilitate comparison, symptom intensity was dichotomized into 0 (no adverse events) and 1 (at least low‐intensity adverse events). For each adverse event we calculated the proportion of children who experienced the event at least once, with 95% Wilson confidence intervals, and compared treatment groups with the χ^2^ test (Yates' correction was applied). In addition, we also graphically presented the adverse events incidence over time for each treatment group.

The primary outcome was *H. pylori* eradication, assessed in both ITT and PP populations. Because these two analyses are predefined and the PP analysis is supportive, no adjustment for multiple testing was applied; a two‐sided α = 0.05 was retained. Adverse‐event analysis was exploratory; *p*‐values are presented unadjusted.

All analyses and visualizations were performed in R version 4.2.2.

## 4 Results

**FIGURE 1:**
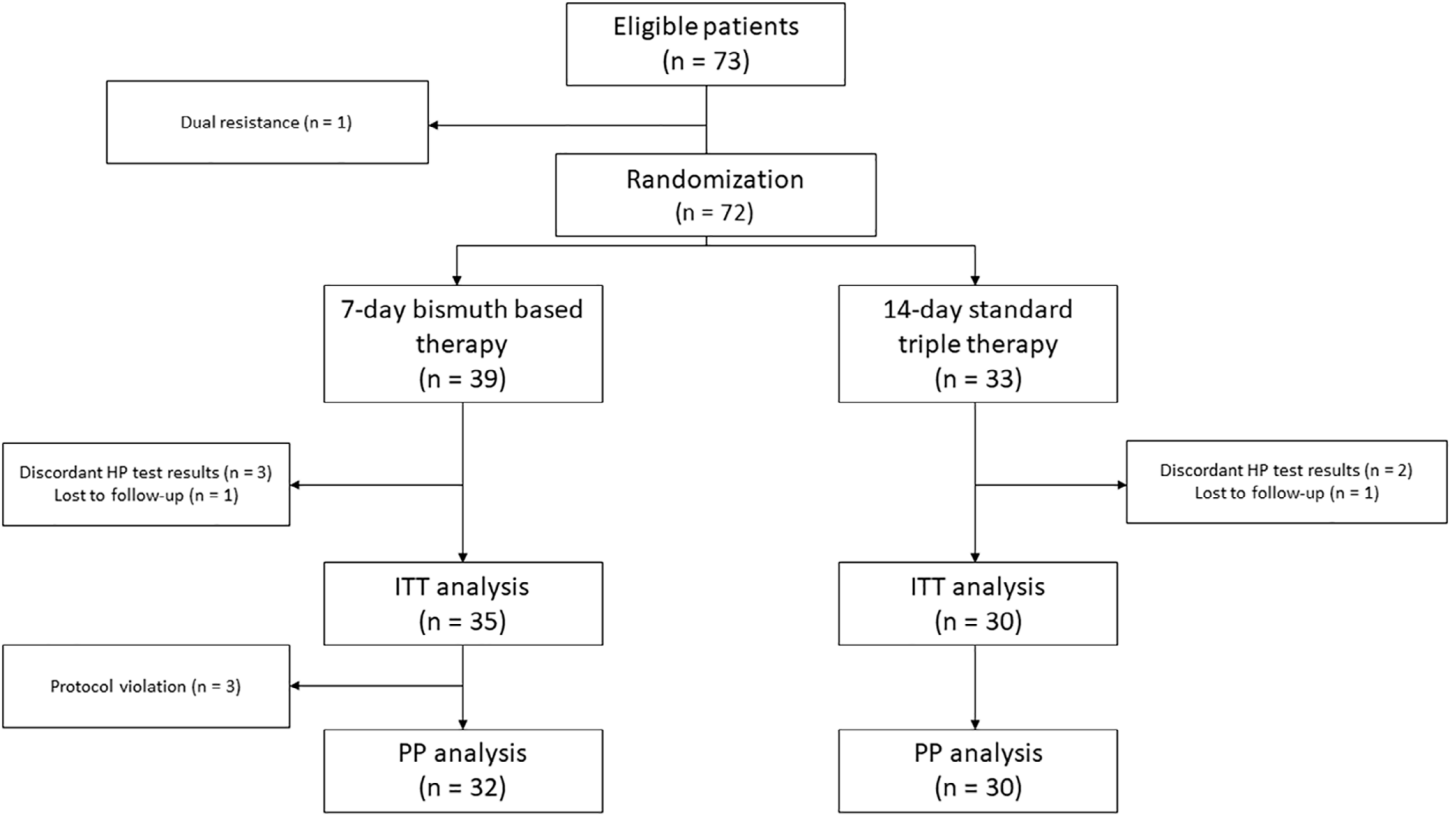
Flowchart of the study. ITT = intention‐to‐treat, PP = per protocol.

A total of 73 children were enrolled during the 4 year study period. One patient was found to be infected with an *H. pylori* strain resistant to both clarithromycin and metronidazole and was therefore excluded from randomization. Thus, 39 and 33 were randomly assigned to the 7 day triple therapy with bismuth and 14 day standard triple therapy groups, respectively (Figure 1).

The basic demographic characteristics of the study participants for both treatment groups are presented in (Table 1). A statistically significant difference was observed between the two groups in the use of susceptibility‐guided antibiotics (clarithromycin vs. metronidazole). CONSORT 2010 advises that covariate adjustment should not hinge on the statistical significance of baseline imbalances, so we followed our pre‐specified analytic plan and did not adjust for this imbalance [14]. For completeness (Table S1), presents sensitivity analyses that adjust for differences in susceptibility‐guided antibiotic use.

**TABLE 1:** Demographic data according to the treatment group.

	Overall	7 day triple therapy with bismuth (*n* = 39)	14 day standard triple therapy (*n* = 33)	*p*
Sex (male), *n* (%)	34 (47%)	20 (51%)	14 (42%)	0.486
Age, median (Q1, Q3)	14.5 (11.7, 16.5)	13.9 (10.0, 16.5)	15.1 (12.4, 16.5)	0.480
Other medications, *n* (%)	9 (13%)	2 (5%)	7 (21%)	0.070
Probiotics, *n* (%)	5 (7%)	2 (5%)	3 (9%)	0.655
CLA (vs MET), *n* (%)	39 (54%)	16 (41%)	23 (70%)	0.019
Full adherence, *n* (%)	62 (86%)	32 (82%)	30 (91%)	0.326

Abbreviations: CLA, clarithromycin; MET, metronidazole; Q, quartile.

(Table 2) presents a comparison of eradication rates between the 7 day triple therapy with bismuth and 14 day standard triple therapy groups, based on both the ITT and PP analyses. In the ITT analysis, four and three participants from the 7 day triple therapy with bismuth and 14 day standard triple therapy groups were excluded due to missing data, respectively (Figure 1). A sensitivity analysis (see Table S2) confirmed that these exclusions did not alter our conclusions. For the PP analysis, an additional three patients were excluded in the group receiving 7 day triple therapy with bismuth due to protocol violation (either early treatment discontinuation or failure to return a diary) and were included in ITT analysis. Although the 7 day triple therapy with bismuth showed a slightly higher eradication rate in both ITT and PP analyses, the differences were not statistically significant—and this finding remained unchanged in the sensitivity analysis (see Table S2).

**TABLE 2:** *Helicobacter pylori* eradication rates according to the treatment group.

	ITT		PP	
	7 day triple therapy with bismuth	14 day standard triple therapy	7 day triple therapy with bismuth	14 day standard triple therapy
Eradication count	32/35	26/30	30/32	26/30
Eradication rate	91%	87%	94%	87%
95% CI	(78%–97%)	(70%–95%)	(80%–98%)	(70%–95%)
Fisher test (*p*)	0.695		0.418	
Risk difference (95% CI)	4.8 (−10.5–20.0)		7.1 (−7.7–21.9)	
Odds ratio (95% CI)	1.64 (0.34–8.00)		2.3 (0.39–13.6)	

Abbreviations: CI, confidence interval; ITT, intention‐to‐treat; PP, per‐protocol.

**FIGURE 2:**
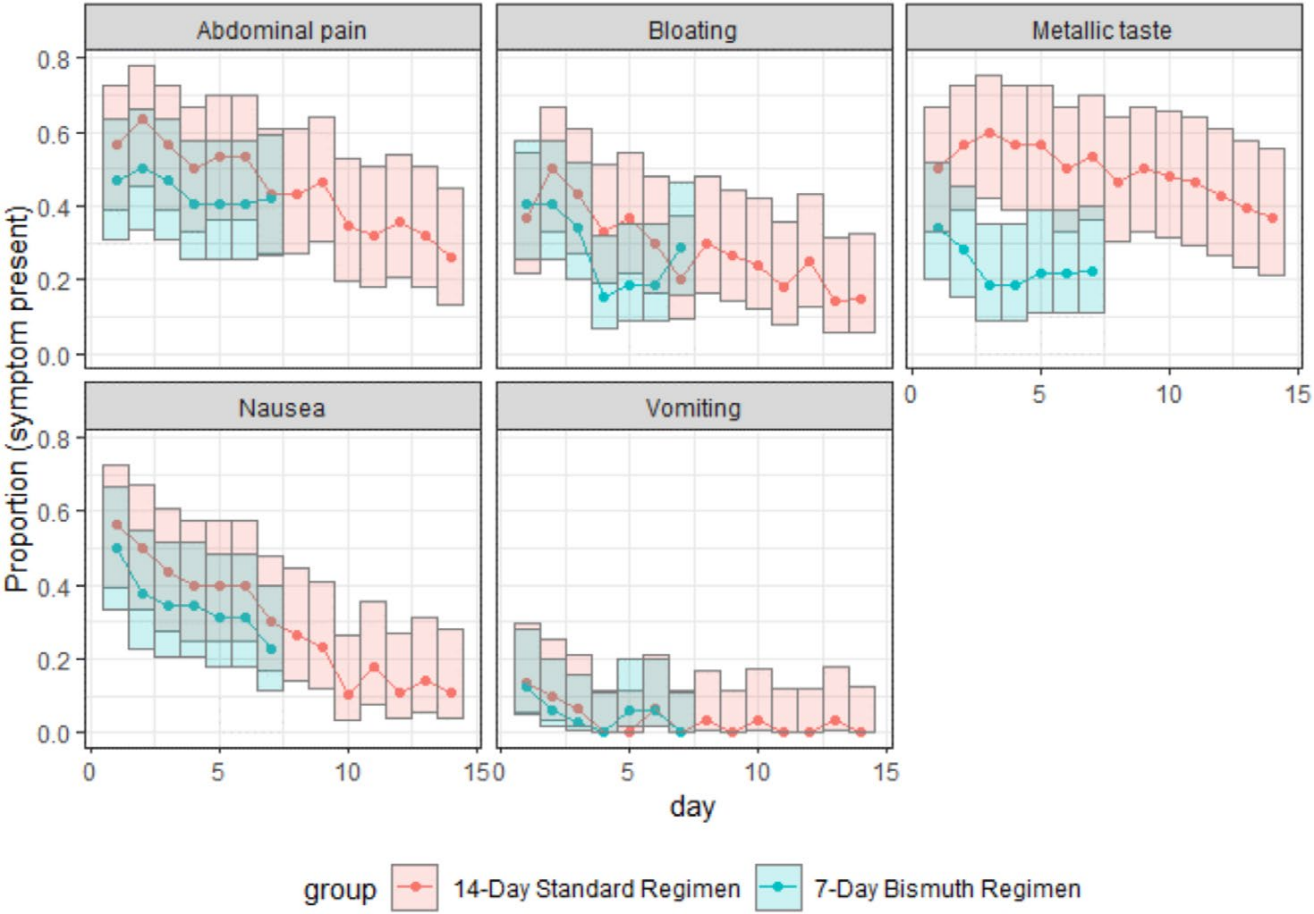
The proportion of patients experiencing adverse events during treatment in each study group, reported with 95% confidence intervals.

No serious adverse events were observed during the study. As shown in Table 3, the incidence of abdominal pain, bloating, nausea, vomiting, diarrhea, and constipation was comparable between treatment groups. However, a significantly higher frequency of metallic taste was reported in the group receiving the 14 day standard triple therapy. In both groups, all adverse events were predominantly of mild to moderate intensity on our sample (data not shown). Other adverse events were more frequently reported in the 14 day standard triple therapy group. Whereas headache was the most frequently reported adverse event in the 14 day standard triple therapy group, a change in stool color was the most observed adverse event in the 7 day study group (Table S3). Lastly, we evaluated how the incidence of adverse events changed during treatment (Figure 2). A decreasing trend was observed for all adverse events, with the most pronounced decline seen in nausea.

**TABLE 3:** Incidence of adverse events reported at least once during the first 7 days of treatment in each treatment group.

Adverse event	7 day triple therapy with bismuth	14 day standard triple therapy	χ^2^ test (*p*)
Abdominal pain	77% (60%–89%)	80% (63%–90%)	1.000
Bloating	58% (41%–74%)	57% (39%–73%)	1.000
Metallic taste	42% (26%–59%)	77% (59%–88%)	0.013
Nausea	61% (44%–76%)	70% (52%–83%)	0.655
Vomiting	19% (9%–36%)	30% (17%–48%)	0.504
Constipation	53% (36%–69%)	47% (30%–64%)	0.799
Diarrhea	44% (28%–61%)	30% (17%–48%)	0.391

## 5 Discussion

This study, conducted in a cohort of treatment‐naïve children, observed higher eradication rates with a 7 day triple therapy with bismuth (ITT: 91%; PP: 94%) compared to the 14‐day standard triple therapy (ITT and PP: 87%). Nevertheless, this difference was not statistically significant, and therefore no conclusion about the superiority of the 7 day triple therapy with bismuth can be made. Nevertheless, superior eradication rates of bismuth‐based therapeutic regimens have been shown previously both in children and adults [4, 15, 16]. The low success rates of standard triple therapy for *H. pylori* eradication are primarily attributed to the increasing resistance to macrolides, and to a lesser extent, imidazoles. Two large multicenter studies from the EuroPedHP registry highlighted the critical role of antibiotic resistance in determining *H. pylori* treatment success in children [17, 18]. The first study, performed between 2013 and 2016, found high regional variability in primary resistance to clarithromycin (25%) and metronidazole (21%), with eradication success of only 79.8% using 7–14 day susceptibility‐guided triple therapy [17]. The second study, conducted between 2017 and 2020, reported even higher rates of primary resistance to clarithromycin (28.8%), yet achieved improved eradication rates (≥ 90%) using 14 day tailored triple therapy in treatment‐naïve patients [18]. Similarly, a 2017 study of 107 treatment‐naïve Slovenian children found high primary resistance to clarithromycin (23.4%) and metronidazole (20.2%), with dual resistance detected in 11.5% of patients [12]. However, in contrast to the findings by Le Thi et al. [18], eradication rates in the Slovenian cohort did not exceed 90%, even when standard triple therapy was tailored to antimicrobial susceptibility [12].

Notably, in settings with high clarithromycin resistance (≥ 15%) or among patients with confirmed clarithromycin‐resistant infections, bismuth‐containing regimens outperform non‐bismuth‐based therapies. The principal advantage of bismuth‐based therapy is its sustained efficacy independent of antibiotic resistance patterns, making it a robust treatment option even in the presence of multidrug‐resistant *H. pylori* strains, whereas dual resistance to clarithromycin and metronidazole significantly compromises the effectiveness of all non‐bismuth‐based regimens [15, 19, 20]. For example, a Spanish study, despite its small sample size, showed that all six children with dual resistance to clarithromycin and metronidazole achieved eradication following treatment with bismuth‐based therapy [21]. Furthermore, data from the Belgian study also demonstrated that a 10 day bismuth‐based regimen was highly effective in children infected with clarithromycin‐resistant or dual‐resistant (clarithromycin and metronidazole) *H. pylori* strains [5].

Although evidence indicates that antimicrobial susceptibility‐guided therapy is generally more effective than empirical treatment, the routine use of pre‐treatment susceptibility testing remains limited, primarily due to practical challenges associated with implementing either culture‐based methods—which are slow and technically demanding due to the fastidious nature of *H. pylori* —or molecular‐based techniques, which may not be readily available in routine clinical settings [22]. According to the latest ESPGHAN/NASPGHAN guidelines for the management of *H. pylori* infection in children and adolescents [6], bismuth‐based therapy is preferred over standard triple therapy where available, due to its higher eradication rates. Moreover, it is recommended as an empiric first‐line treatment in children in the absence of antimicrobial susceptibility testing. Although the precise mechanism by which bismuth compounds exert antibacterial activity against *H. pylori* is not fully understood, they appear to have a direct antimicrobial effect and can disrupt the bacterium's ability to adhere to the gastric epithelium [23, 24]. Moreover, *H. pylori* strain resistant to bismuth has not yet been described [24], further supporting its use where available.

The optimal duration of *H. pylori* eradication therapy has been a subject of ongoing debate. While previously a 7 day triple therapy had been recommended, a Cochrane meta‐analysis of 55 studies revealed its relatively low eradication rate of 72.9%, compared to 81.9% with a 14 day regimen [25]. These findings prompted recommendations for extending treatment to at least 10–14 days, despite the potential for increased adverse events and reduced patient adherence [9, 10]. A recently published multicenter, randomized, non‐inferiority trial conducted in Taiwanese adults compared 10 day and 14 day bismuth‐based regimens as first‐line therapy for *H. pylori* eradication. The study demonstrated that the shorter regimen was non‐inferior to the standard 14‐day treatment, with eradication rates of 92.4% vs. 92.9% by ITT analysis and 97.9% vs. 99.3% by PP analysis [26]. Similarly, shorter‐duration bismuth‐based regimens proved effective in achieving high eradication rates also among children. In a study by Kotilea et al. [5] involving Belgian children aged 6–17 years, a 10 day bismuth‐based therapy, comprising colloidal bismuth subcitrate, a PPI, amoxicillin, and metronidazole achieved eradication in 35 of 36 patients (95% CI: 85%–99%). Moreover, a 7‐day bismuth‐based therapy significantly outperformed the 2‐week standard triple therapy in eradicating *H. pylori* among Korean children (83.9% vs. 67.7%; *p* = 0.041) [27]. Reducing the duration of eradication therapy may improve compliance not only by lowering treatment costs and the risk of adverse events, but also because shorter regimens are easier for patients to complete. Specifically, a pediatric cohort study from Belgium demonstrated that eradication rates were significantly higher among children with high adherence to therapy (> 90%), which was more commonly observed in children without chronic illnesses or adverse events [7]. A recent systematic review and meta‐analysis of four studies involving 1173 patients found that 10‐day and 14‐day bismuth‐based therapy had comparable adherence and eradication rates (risk ratio [RR] 0.97, 95% CI: 0.93–1.01) in the ITT analysis, while the 10‐day regimen was associated with fewer adverse events [28]. Similarly, in a study by Yang et al. [26], adult patients who received a 10‐day bismuth‐based eradication therapy had significantly lower rates of dizziness (18.5% vs. 34.0%) and vomiting (4.5% vs. 12.8%) than those receiving a 14 day therapy, although the overall incidence of adverse events did not differ between the two groups. In contrast, two studies conducted among Korean adults with *H. pylori* infection reported no statistically significant difference in the prevalence of adverse events between 7 day and 14 day bismuth‐based therapy regimens [29, 30].

In this study, adverse events related to *H. pylori* eradication treatment were predominantly gastrointestinal, mild in intensity, and occurred at comparable rates between the two treatment groups. These findings are consistent with previous studies demonstrating the favorable safety and tolerability profile of bismuth‐containing regimens in pediatric populations [4, 16]. In a study involving treatment‐naïve Chinese children who were randomized to receive either standard triple therapy, sequential therapy, bismuth‐based therapy, or concomitant therapy, the most frequently reported adverse events were abdominal discomfort, nausea, and vomiting, which occurred at rates comparable across all treatment regimens [4]. Similarly, a study conducted in Vietnam involving 160 children with peptic ulcer disease receiving a bismuth‐based regimen identified nausea and vomiting (19.9%) as the most frequently reported adverse events, followed by fatigue (11.8%), headache (3.7%), and diarrhea (0.7%). Importantly, all adverse events were mild, self‐limiting, and did not lead to treatment discontinuation [16]. Previously, concerns have been raised regarding the palatability of bismuth potentially affecting adherence [8]; however, none of the patients assigned to the 7 day triple therapy with bismuth group reported issues with the palatability of bismuth in this study. Interestingly, in our study, adverse events such as headache and metallic taste were more commonly reported among children receiving the 14 day standard triple therapy, further supporting the favorable safety profile of bismuth‐containing *H. pylori* eradication regimens in pediatric patients. In addition, it has been previously shown that a 14 day bismuth‐based therapy, comprising a PPI, bismuth, clarithromycin, and metronidazole, induces transient dysbiosis of the gut microbiota, with most alterations resolving within one year post‐eradication, thereby supporting the long‐term safety of *H. pylori* treatment in children [31].

The primary limitation of the study was the relatively small sample size. Several factors contributed to the limited number of enrolled children. First, a significant proportion were ineligible due to recent use of PPIs or antibiotics. Second, several participants were excluded during the study due to protocol violations or inconclusive post‐eradication test results (Figure 1). As the final sample size was smaller than the a priori target, the study was underpowered to detect modest between‐group differences. Nonetheless, reporting these findings is justified given the scarcity of pediatric *H. pylori* data, their relevance for clinical practice, and their potential value for future multicenter studies and meta‐analyses.

## 6 Conclusions

Taken together with existing evidence in pediatric and adult populations, our findings support the 7 day triple therapy with bismuth as a safe and well‐tolerated option for *H. pylori* eradication in pediatric patients. With an eradication rate exceeding 90%, which was slightly higher than the eradication rate of the 14 day standard triple therapy, the shorter, 7 day triple therapy with bismuth appears clinically rational, supports the principles of antimicrobial stewardship, reduces costs, and may enhance adherence, particularly in pediatric populations. To validate the results of our study, a multi‐centric pediatric study should be performed with a higher number of included children.

## Funding

This work was supported by Javna Agencija za Raziskovalno Dejavnost RS, P3‐0154.

## Supporting information


**Table S1:** Odds ratio and 95% confidence intervals for the Firth's penalized‐likelihood logistic regression for ITT and PP samples.
**Table S2:** Sensitivity analysis of *H. pylori* eradication rates by treatment group under best‐ and worst‐case scenarios.
**Table S3:** Other adverse events reported by the patients during treatment.

## Data Availability

The data that support the findings of this study are openly available in [repository name] at [DOI].
