# Supplementary material for: An Open‐Label Randomized Controlled Trial Comparing the Efficacy and Safety of a 7‐Day Triple Therapy With Bismuth Versus 14‐Day Standard Triple Therapy for Helicobacter pylori Eradication in Children and Adolescents
Source: Helicobacter. 2026 Jan 7;31(1):e70103. doi: 10.1111/hel.70103 (PMC12776654; doi:10.1111/hel.70103)
Supplement: Supplementary file 1 — Table S1: Odds ratio and 95% confidence intervals for the Firth's penalized‐likelihood logistic regression for ITT and PP samples. Table S2: Sensitivity analysis of H. pylori eradication rates by treatment group under best‐ and worst‐case scenarios. Table S3: Other adverse events reported by the patients during treatment. [file HEL-31-e70103-s001.docx]

**Supplementary material**

**An open-label randomized controlled trial comparing the efficacy and safety of a 7-day triple therapy with bismuth versus 14-day standard triple therapy for *Helicobacter pylori* eradication in children and adolescents**

**Anja Šterbenc^1^, Bor Vratanar^2^, Eva Miler Mojškerc^3^, Matjaž Homan^4^**

Content

Table S1. Firth’s penalized‐likelihood logistic regression Page 2

Table S2. Sensitivity analysis for missing eradication status Page 3

Table S3. Other adverse events Page 4

**Sensitivity analysis**

We conducted a sensitivity analysis using Firth’s penalized‐likelihood logistic regression to assess whether baseline differences in susceptibility-tailored antibiotics between the two groups influenced our findings. We fitted two models with eradication status as the outcome (0 = not eradicated, 1 = eradicated). The first model included only the susceptibility-tailored antibiotic, and the second model additionally included a treatment group. We then compared the models using a penalized likelihood‐ratio test. In both the ITT and PP samples, adding treatment group did not significantly improve the model fit (ITT: χ^2^ = 2.45, df = 1, p = 0.117; PP: χ^2^ = 3.14, df = 1, p = 0.077). Thus, adjustment for susceptibility-tailored antibiotic regimen did not affect our conclusions. The full Firth‐corrected estimates for both covariates are presented in Table S1. In our sample, after adjusting for susceptibility-tailored antibiotic, point estimates suggest a higher eradication rate with the 7-day bismuth-based therapy than with the 14-day standard triple therapy.

**Table S1. Odds ratio and 95% confidence intervals for the Firth’s penalized‐likelihood logistic regression for ITT and PP samples.**

|  | ITT | |  | PP | |
| --- | --- | --- | --- | --- | --- |
|  | Odds ratio | 95% CI |  | Odds ratio | 95% CI |
| Susceptibility-tailored antibiotic^a^ | 10.5 | 1.7 to 119.6 |  | 10.0 | 1.5 to 115.7 |
| Group^b^ | 3.8 | 0.7 to 22.8 |  | 5.0 | 0.8 to 36.6 |

^a^ 0 = metronidazole, 1 = clarithromycin.

^b^ 0 = 14-day standard triple therapy, 1 = 7-day bismuth- based therapy.

ITT = intention-to-treat, PP = per-protocol, CI = confidence interval.

We also conducted a sensitivity analysis on the ITT population to assess the impact of missing eradication outcomes (four in the 7-day bismuth group and three in the 14-day standard triple group). We applied two extreme scenarios: in the best-case scenario, all missing values in the bismuth group were assumed eradicated and all missing values in the triple-therapy group were assumed not eradicated. In the worst-case scenario, the assumptions were reversed. Under both scenarios, the difference in *H. pylori* eradication rates between the two treatment arms remained statistically insignificant.

**Table S2. Sensitivity analysis of *H. pylori* eradication rates by treatment group under best- and worst-case scenarios.**

|  | ITT - best | |  | ITT - worst | |
| --- | --- | --- | --- | --- | --- |
|  | 7-day bismuth- based therapy | 14-day standard triple therapy |  | 7-day bismuth- based therapy | 14-day standard triple therapy |
| Eradication count | 36 / 39 | 26 / 33 |  | 32 / 39 | 29 / 33 |
| Eradication rate  (95 % CI) | 92%  (80% to 97%) | 79%  (62% to 89%) |  | 82%  (67% to 91%) | 88%  (73% to 95%) |
| Fisher test  *(p*-value) | 0.170 | |  | 0.533 | |
| Risk difference  (95 % CI) | 13.5 (-2.7 to 29.8) | |  | -5.8 (-22.2 to 10.6) | |
| Odds ratio  (95 % CI) | 3.23 (0.76 to 13.68) | |  | 0.63 (0.17 to 2.38) | |

ITT = intention-to-treat, PP = per-protocol, CI = confidence interval.

**Table S3. Other adverse events reported by the patients during treatment.**

| Adverse event | 7-day bismuth-based therapy  (n = 39) | 14-day standard triple therapy  (n = 33) |
| --- | --- | --- |
| Headache | 0 | 6 (18%) |
| Change in stool color | 3 (8%) | 0 |
| Blood in stools | 0 | 1 (3%) |
| Tongue rash | 0 | 1 (3%) |
| Skin rash | 3 (8%) | 1 (3%) |
| Increased salivation | 1 (3%) | 0 |
| Dizziness | 0 | 2 (6%) |
| Burning sensation in mouth | 1 (3%) | 0 |
| Mild tremor | 0 | 1 (3%) |
| Hiccups | 0 | 1 (3%) |
| All adverse events | 8 (21%) | 13 (39%) |
